# Effects of an exercise program on hepatic metabolism, hepatic fat, and cardiovascular health in overweight/obese adolescents from Bogotá, Colombia (the HEPAFIT study): study protocol for a randomized controlled trial

**DOI:** 10.1186/s13063-018-2721-5

**Published:** 2018-06-25

**Authors:** Katherine González-Ruíz, Jorge Enrique Correa-Bautista, Mikel Izquierdo, Antonio García-Hermoso, María Andrea Dominguez-Sanchez, Rosa Helena Bustos-Cruz, Jorge Cañete García-Prieto, Vicente Martínez-Vizcaíno, Felipe Lobelo, Emilio González-Jiménez, Daniel Humberto Prieto-Benavides, Alejandra Tordecilla-Sanders, Jacqueline Schmidt-RioValle, Guillermo Perez, Robinson Ramírez-Vélez

**Affiliations:** 1 Centro de Estudios en Medición de la Actividad Física (CEMA), Escuela de Medicina y Ciencias de la Salud, Universidad del Rosario, Cra. 24 No. 63C – 69, Bogotá, DC Colombia; 2 Grupo de Ejercicio Físico y Deportes, Facultad de Salud, Programa de Fisioterapia, Universidad Manuela Beltrán, Bogotá, DC Colombia; 3 Department of Health Sciences, Public University of Navarra, Pamplona, Navarra Spain; 4 Laboratorio de Ciencias de la Actividad Física, el Deporte y la Salud, Facultad de Ciencias Médicas, Universidad de Santiago de Chile, USACH, Santiago, Chile; 5 Grupo de Investigación Movimiento Corporal Humano, Facultad de Enfermería y Rehabilitación, Universidad de La Sabana, Chía, Colombia; 6 Evidence-Based Therapeutic Group, Clinical Pharmacology, Universidad de la Sabana, Bogotá, DC Colombia; 7 MOVI-FITNESS Research-based company (Spin-Off), Universidad de Castilla-La Mancha, Cuenca, Spain; 8 Health and Social Research Center, Universidad de Castilla-La Mancha, Cuenca, Spain; 9 Facultad de Ciencias de la Salud, Universidad Autónoma de Chile, Talca, Chile; 10 Hubert Department of Global Health, Rollins School of Public Health and Exercise is Medicine Global Research and Collaboration Center, Emory University, Atlanta, GA USA; 11 Departamento de Enfermería, Facultad de Ciencias de la Salud, University of Granada, Avda. De la Ilustración, 60, 18016 Granada, Spain; 12 Serviendoscopias Unidad de Gastroenterología y Endoscopia, Bogotá, DC Colombia

**Keywords:** Exercise, Risk factor, Fatty liver, Metabolic syndrome, Overweight, Obesity

## Abstract

**Background:**

A considerable proportion of contemporary youth have a high risk of obesity-related disorders such as cardiovascular disease, metabolic syndrome, or non-alcoholic fatty liver disease (NAFLD). Although there is consistent evidence for the positive effects of physical activity on several health aspects, most adolescents in Colombia are sedentary. It is, therefore, important to implement strategies that generate changes in lifestyle. The HEPAFIT study aims to examine whether a 6-month exercise program has benefits for hepatic fat content and cardiovascular health outcomes among overweight/obese adolescents from Bogotá, Colombia.

**Methods/design:**

Altogether, 100 hundred overweight/obese, sedentary adolescents (aged 11–17 years) attending two public schools in Bogotá, Colombia, will be included in a parallel-group randomized controlled trial. Adolescents will be randomly assigned to an intervention group following one of four curricula: (1) the standard physical education curriculum (60 min per week of physical activity, *n* = 25) at low-to-moderate intensity; (2) a high-intensity physical education curriculum (HIPE, *n* = 25), consisting of endurance and resistance games and non-competitive activities, such as running, gymkhanas, lifting, pushing, wrestling, or hauling, for 60-min sessions, three times per week, with an energy expenditure goal of 300 to 500 kcal/session at 75–85% maximum heart rate (HRmax); (3) a low-to-moderate intensity physical education curriculum (LIPE, *n* = 25) consisting of endurance and resistance games and non-competitive activities (e.g., chasing, sprinting, dribbling, or hopping) for 60-min sessions, three times per week with an energy expenditure goal of 300 kcal/session at 55–75% HRmax; and (4) a combined HIPE and LIPE curriculum (*n* = 25). The HIPE, LIPE, and combined interventions were performed in addition to the standard physical education curriculum. The primary outcome for effectiveness is liver fat content, as measured by the controlled attenuation parameter 1 week after the end of the intervention program.

**Discussion:**

The translational focus may be suitable for collecting new information in a school setting on the possible effects of physical activity interventions to reduce liver fat content and to improve metabolic profiles and the cardiometabolic health of overweight/obese adolescents. This may lead to the more efficient use of school physical education resources.

**Trial registration:**

ClinicalTrials.gov, NCT02753231. Registered on 21 April 2016.

**Electronic supplementary material:**

The online version of this article (10.1186/s13063-018-2721-5) contains supplementary material, which is available to authorized users.

## Background

Pediatric non-alcoholic fatty liver disease (NAFLD) is considered the hepatic manifestation of metabolic syndrome and is the most common early driver of chronic liver disease in the United States [1]. However, the true prevalence of pediatric NAFLD is still not known for the following reasons: (i) to date, only a few population studies have been conducted in pediatric populations, (ii) different screening or diagnostic methods were used, and (iii) sex and ethnic differences were also reported [2]. In obese child populations, the prevalence of NAFLD ranges between 7.6% and 34.2% [3]. Moreover, the prevalence of NAFLD has increased in parallel with the prevalence of obesity, metabolic syndrome, and type 2 diabetes [2]. There is an association between obesity, especially abdominal obesity, with visceral adipose tissue and hepatic fat. Obesity induces metabolic disorders, such as NAFLD, through diverse pathways that involve adipocytokines, insulin and leptin resistance, overproduction of free radicals, and inflammation of adipose and liver tissue. If body fat is accumulated predominately in the upper body or abdominal cavity, the fat is mainly visceral, which has a greater impact on liver fat content and also positively correlates with the potential for developing NAFLD [4, 5].

The metabolic complexity of NAFLD presents challenges for a deeper understanding of the metabolic pathways that contribute to the development of this pathology [6, 7]. In the last few years, a growing number of studies have demonstrated that non-traditional biomarkers (Additional file 1: Table S1) that function as regulators of metabolic function may be involved in the etiopathogenesis of NAFLD and other metabolic disorders [8, 9]. Progress may be aided by a comprehensive metabolic analysis of the condition before and after the disease is established. Thus, several studies have observed that oxidative stress, inflammation, and insulin resistance are implicated in several aspects of glucose homeostasis and lipid metabolism and are associated with the pathogenesis and progress of NAFLD [10]. Moreover, non-traditional biomarkers may be used to assess biological adaptations to physiological changes or environmental exposures, such as lifestyle intervention programs based on increasing physical activity levels, improving dietary habits, and promoting a healthy weight. Likewise, the measurement of non-traditional and classical biomarkers, such as hepatic metabolism and cardiovascular biomarkers, presents an opportunity to evaluate biological changes associated with lifestyle interventions to reduce metabolic disorders associated with NAFLD progression, such as oxidative stress, inflammation, and insulin resistance.

There is evidence that sedentary lifestyles and unhealthy diets (especially those that are high in fats, free sugars, and salt) are driving the obesity epidemic and its co-morbidities in pediatric populations [11]. However, no medications or surgical procedures have been approved for the treatment of NAFLD. In turn, regular physical exercise has been shown to promote positive adaptations among obese children and adolescents [12]. Currently, there are no dietetic or physical activity guidelines for the management of NAFLD in children and youth. However, practice guidelines from the American Association for the Study of Liver Diseases have recommended lifestyle interventions to reduce body weight by 7–10% as the primary prevention and management of NAFLD in adults [13]. A recent meta-analysis in an obese pediatric population supports the current recommendation for physical exercise, mainly aerobic, as an effective intervention to counteract NAFLD progression by targeting hepatic lipid composition, visceral adipose tissue, and subcutaneous adipose tissue [14]. Still, the optimal exercise type (aerobic, resistance, or combined exercise training) and intensity (moderate or vigorous) to slow NAFLD progression in young populations is unclear.

Previous systematic reviews and meta-analyses in obese children and adolescents have shown that exercise reduces cardiometabolic risk factors, such as visceral adiposity [14], insulin resistance [12], inflammatory biomarkers [15], and hepatic lipid composition [14]. In addition, a growing body of evidence has demonstrated comparable or superior improvements in cardiometabolic health outcomes using low-volume, high-intensity interval training (HIIT) compared with traditional moderate-intensity continuous training (MICT) in adults [16] and the young population [17]. In a clinical setting, HIIT is an effective way of performing high-intensity exercise training and developing a high level of cardiorespiratory fitness [16]. A recent meta-analysis of 28 trials confirmed that both MICT and HIIT elicit large improvements in peak VO_2_ of healthy, young to middle-aged adults, with the effects being greater for the less fit [16]. Moreover, few HIIT interventions in children or adolescents have studied the effects on hepatic adiposity parameters, so further research is required.

Recently, Medrano et al. [18] proposed that a multidisciplinary intervention program including education healthy lifestyles, psychoeducation, and supervised exercise could play a key role in reducing hepatic fat and cardiometabolic risk in overweight children. In this context, four previous studies in children and adolescents examined the effect of supervised exercise without caloric restriction on hepatic fat [19–22]. However, the majority of the studies do not aim to identify non-traditional biomarkers as the surrogate end point of NAFLD. Previous reports observed changes in some circulating traditional biomarkers after liraglutide therapy [23] or exercise in NAFLD adults [24]. In obese youth, several P-selectins, endocan [25], insulin-like growth factor 1 [26], and some proinflammatory cytokines [27] have been proposed as biomarkers of endothelial dysfunction, dyslipidemia, inflammation, insulin resistance, and NAFLD. As far as we are aware, there are no previous studies identifying the profile of non-traditional biomarkers in adolescents at a high risk of NAFLD and the response to an intervention with exercise.

Given that physical exercise increases insulin sensitivity, increases oxidative metabolic activity, and reduces hepatic fat and cardiometabolic risk in a young obese population, the HEPAFIT study aims to examine whether a 6-month exercise program has benefits for hepatic fat content and inflammation as well as cardiovascular health outcomes among adolescents from Bogotá, Colombia, who are overweight or obese.

## Methods/design

### Study design, setting, and participants

The HEPAFIT study is a single-blind parallel-group randomized controlled trial (ClinicalTrials.gov ID NCT02753231). All parents or guardians will provide informed written consent and all the adolescents must give assent before being enrolled in the study. The study received ethical approval from the Medical Research Ethics Committee of the University of Rosario (ID UR-21042016). Random allocation to treatments will be performed at the individual level.

The study will include 100 adolescents (50% females) between 11 and 17 years of age attending to two public schools in the urban area of Bogotá D.C., Colombia. Inclusion criteria include primary overweight/obese status, defined according to the International Obesity Task Force [28], inactivity (no habitual participation in exercise more than once a week in the previous 6 months), and having at least one parent or caregiver willing to participate in the program sessions. Adolescents eligible for the present study will be invited to a pre-test, which will include a medical interview at the Centre for Studies of Physical Activity Measurements (in Spanish, CEMA), School of Medicine and Health Sciences, University of Rosario, Bogotá, Colombia. Risks will be minimized by identifying contraindications to the testing and training protocols via a health history and a thorough physical examination prior to the testing sessions. The exclusion criteria include having a clinical diagnosis of cardiovascular disease, having type 1 or type 2 diabetes mellitus, being pregnant, using alcohol or drugs, and not having lived in Bogotá for at least one school year. Adolescents with other causes of liver disease in the pediatric population that produce elevated liver enzyme levels will be excluded. These will be identified by screening for hepatotropic viruses (hepatitis A, B, and C, cytomegalovirus, and Epstein–Barr virus) and alpha-1 antitrypsin (A1AT) levels as well as autoantibodies and immunoglobulin G to rule out A1AT deficiency and autoimmune hepatitis, respectively. This study aims to investigate the effects of these interventions on risk factors for type 2 diabetes (i.e., its focus is prevention) rather than looking at their effects on treatment outcomes. Therefore, participants with established type 2 diabetes are excluded.

### Recruitment

Consecutive boys or girls with overweight/obesity will be recruited from two public schools if they have received referrals from physician to either a primary- or secondary-care general practitioner in the capital district of Bogotá, Cundinamarca Department, in the Andean region. After completion of the informed consent process, adolescents who are interested in participating will be approached with further information and undergo pre-participation screening to determine contraindications to exercise using a cardiovascular and musculoskeletal checklist (including the patient’s medical history, disease history, and physical fitness). A member of the research team will follow up with a phone call to screen for eligibility and explain the main requirements of the study.

### Blinding and randomization methods

**Fig. 1:**
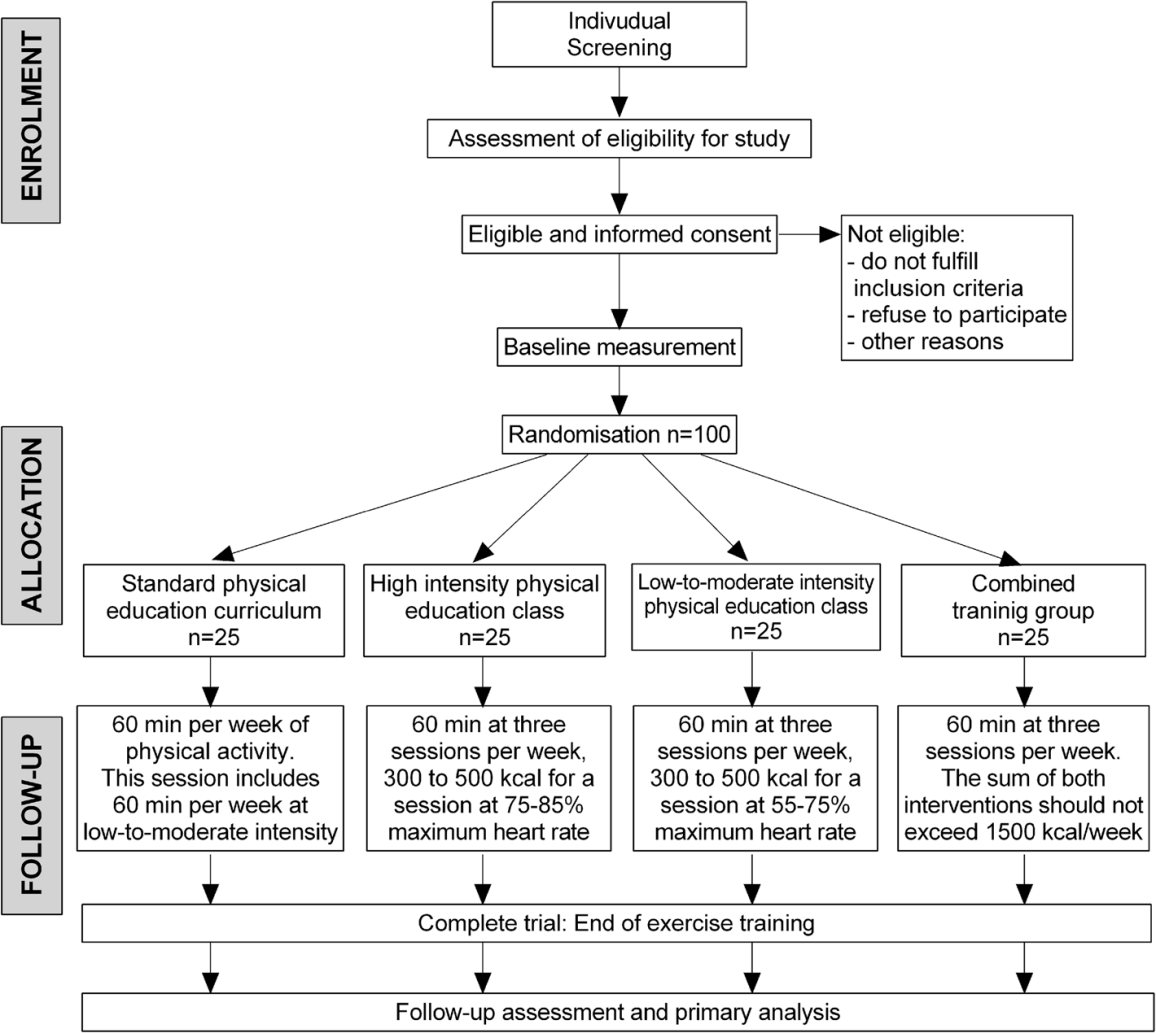
CONSORT guidelines flow diagram for enrolment and randomization HEPAFIT Study

The randomization into the four study arms will be performed by CEMA using a block randomization strategy with a block size of four. Since growing adolescents have an increasing body size and are undergoing developmental changes, a control group is necessary to detect changes due to training. After completing the baseline measurements, eligible participants will be randomly assigned to either the control or exercise training groups. The principal investigator will coordinate the allocation sequence, and randomization will be computer-generated. All participants and study personnel (including investigators, trainers, and statisticians) will be blinded to treatment allocation throughout the trial protocol. Access to the allocation code will be restricted to one study statistician who will not perform the final study analyses. Randomization will be conducted independently using sealed opaque envelopes. These procedures are also detailed in the study operations manual. Moreover, the importance of maintaining the blinding and allocation concealment will be reinforced by regularly scheduled conference calls at the sites and daily meetings with the field investigators (Fig. 1).

### Intervention

Participants will be randomly assigned to the following groups.

#### Standard physical education curriculum

This is the control group (*n* = 25). These subjects will not carry out any intervention different from the physical education class prescribed by the Colombian education legislation (Legislation 115 of 1994, decree 1860 of 1994) [29], which is recognized as fundamental and is consequently obligatory in all educational institutions in the country. This session includes 60 min per week of physical activity at low-to-moderate intensity. However, during this period, education will be offered in lifestyles, hygiene, and diet [30] at no cost to participants. Nutritional counseling will be offered as well.

#### High-intensity physical education class

The high-intensity physical education group (HIPE group, *n* = 25) will follow the standard physical education curriculum plus the Physical Activity Intervention on the Obesity of Schoolchildren (in Spanish, the MOVI-2 program), which is based on a socio-ecological model and targets the school, parents, teachers, and children, focusing on increasing physical activity [31]. MOVI-2 includes basic sports games, traditional games, and other outdoor activities such as running, gymkhanas (which consist of different types of competition with obstacles that make the task difficult), lifting, pushing, wrestling, and hauling (http://www.movidavida.org/), for 60-min sessions three times per week for 6 months with an energy expenditure goal of 300 to 500 kcal/session at 75–85% maximum heart rate (HRmax).

For example, the prescribed energy expenditure during exercise for month 1 is 300 kcal session^− 1^:

1. Conveyors game: Exercise energy expenditure is 9.2 kcal min^− 1^ and exercise duration is 100 kcal per session. Dividing by 9.2 kcal min^− 1^ gives 11 min per session.
2. Four corners game: Exercise energy expenditure is 10.8 kcal min^− 1^ and exercise duration is 100 kcal per session. Dividing by 10.8 kcal min^− 1^ gives 9 min per session^.^
3. Stealing handball game: Exercise energy expenditure is 9.4 kcal min^− 1^ and exercise duration = 100 kcal per session. Dividing by 9.4 kcal min^− 1^ gives 10 min per session.

All playground games will be preceded by a 5-min warm up with endurance or resistance games (i.e., the fastest, the clock, iceman, kangaroo race, etc.). The duration and intensity of all playground games sessions will be verified by a downloadable heart rate (HR) monitor (RS 400; Polar Electro Inc., Woodbury, NY) to achieve the prescribed target HR. All activities will be implemented by monitors with technical qualifications in physical activity and sports, or by sport science graduates, specifically engaged and adequately trained for the program. This protocol is applicable for school-aged boys and girls, independent of the level of skill or their physical aptitude, and is a health-oriented program aimed at balanced physical and psychological development.

The MOVI program uses attractive alternative materials not often used in the subjects’ daily lives or in the standard physical education classes. It is accessible, given that it occurs at the end of the classroom activities in the school’s facilities or nearby. It is free of charge and does not require the provision of any specific equipment. In its first edition, the MOVI program showed a moderate effect on reducing adiposity and improving the lipid profile but did not significantly improve global cardiometabolic risk or lower insulinemia [32]. In the second edition (MOVI-2), the duration and intensity of the sessions were increased and it was shown to enhance the development of muscle strength further. Likewise, analyses showed reduced adiposity in both sexes, and an improvement in the girls’ cardiovascular risk profile (low-density lipoprotein cholesterol or LDL-C and insulin) was observed [33]. The MOVI-2 program sessions that will be used in this work are the following:

Frequency: 3 days per week excluding holidays and vacation periods

Duration: 60 min, structured into 5 min of warm-up, 45 min dedicated to the main training curriculum, and 10 min dedicated to returning to a rest state

Protocol: Each of the sessions will work on activities based on health-related physical fitness (cardiovascular and muscle endurance and flexibility) at an intensity of between 8.0 and 11.7 metabolic equivalents (MET). All the games were performed previously by 32 schoolchildren during sessions of the MOVI-2 program while indirect calorimetry (through a portable gas analyzer), cardiac frequency, and accelerometry were evaluated for each game [34]. In addition, we have previously monitored the implementation of all the games with overweight/obese adolescents from Bogotá (unpublished observations).

#### Low-to-moderate intensity physical education class

The low-to-moderate intensity physical education group (LIPE group, *n* = 25) will follow the standard physical education curriculum. In addition, endurance and resistance games from the MOVI-2 program will be used with a goal to achieve an expenditure of 1500 kcal/week through 60-min sessions, three times per week for 6 months for 300 to 500 kcal energy expenditure per session at 55–75% HRmax:

Frequency: Three times weekly, excluding holidays and vacation periods

Duration: 60 min, structured into 5 min of warm-up, 45 min dedicated to the main training curriculum, and 10 min dedicated to returning to a rest state

Protocol: Each of the sessions will work on activities based on health-related physical fitness (cardiovascular and muscle endurance and flexibility) at an intensity of between 4.0 and 7.0 MET.

#### Plus group

The combined group (*n* = 25) performed the 30-min aerobic training program plus the 30-min resistance training, that involve training program used in both HIPE and LIPE. Each session is developed as described above. Additionally, the standard physical education curriculum was followed. The sum of both interventions should not exceed 1500 kcal/week of energy expenditure. All the study participants in this group will receive a hygiene and dietary management plan that includes educational and nutritional support throughout the study.

After 3 months, the intensity will increase and a different exercise sequence will be used until 6 months of intervention have taken place. The exercise duration will be adjusted to achieve the target average caloric expenditure of 1500 kcal/week. Compliance to the exercise protocol, an essential element of an efficacy study, will be defined as successfully completing >90% of scheduled exercise sessions, maintaining the target exercise HR ± 4 beats min^− 1^ for the prescribed duration of the playground games session. The games proposed for this group are presented in Table 1. It should be noted that diverse variations on the games will be carried out to avoid monotony.

**Table 1:**
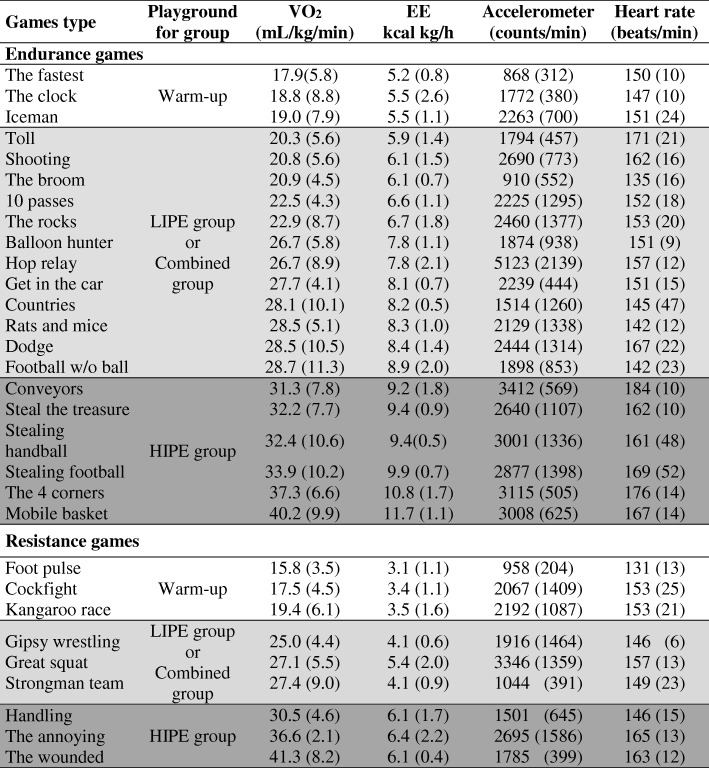
Example of playground games ranked by oxygen consumption and energy expenditure with accelerometer counts and heart rate data also displayed for the HEPAFIT study

*EE* energy expenditure, *HIPE* high-intensity physical education, *LIPE* low-to-moderate intensity physical education

The data for each game is an average for 5 ± 1 subjects (with standard error).  Games with VO_2_ up to 20 mL kg^–1^ min^–1^.  Games with VO_2_ between 20 and 30 mL kg^–1^ min^–1^.  Games with VO_2_ above 30 mL kg^–1^ min^–1^. A full description for the preparation and administration of these games can be found at www.movidavida.org. Table taken from Cañete-García et al. [34]

### Training intensity and energy expenditure during training

Training intensity will be assessed using the mean HR measured in the HIPE, LIPE or combined groups. The exercise training program will be 100% supervised. Attendance at supervised sessions includes compliance with target HR and energy expenditure and will be monitored and recorded by research staff. Energy expenditure will be estimated during exercise via indirect calorimetry using a Cosmed K-5*b*^2^ portable metabolic system (Rome, Italy), assuming a non-protein respiratory exchange ratio [35]. This procedure will be assessed for 30% of participants to help to maintain compliance and to provide a detailed description of each playground games session. It is expected that the gradual increase in total energy expenditure will minimize fatigue, soreness, injuries, and attrition.

### Data collection and outcome measures

**Fig. 2:**
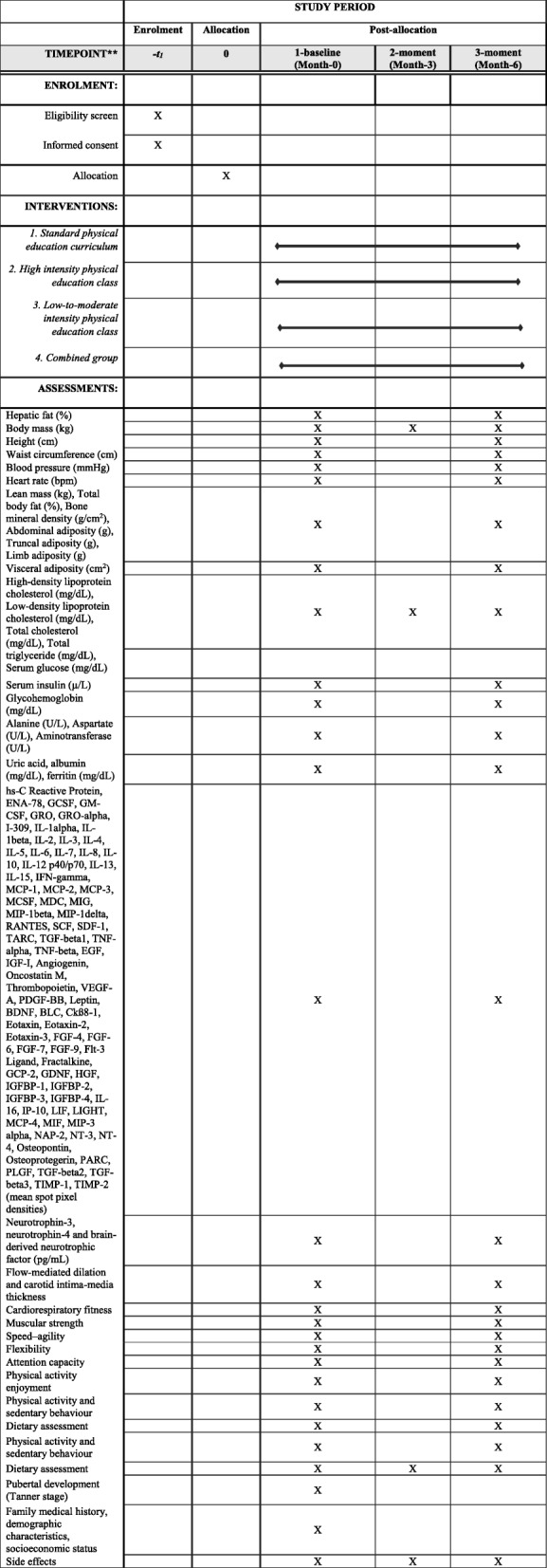
Schedule of enrolment, intervention, and assessment in the HEPAFIT study, according to the SPIRIT 2013 statement

Outcome measures will be assessed at baseline and at the end of the 6-month intervention by personnel blinded to the treatment allocation. Data will be recorded on standardized forms and entered into a secured Access database that contains quality control checks (e.g., range checks and notifications for missing data) (Fig. 2). Post-intervention measurements will be scheduled within 7 days following the last exercise session or last supervised program session in the exercise and control groups, respectively.

The primary outcome measure is hepatic fat content, which will be assessed with the controlled attenuation parameter. The secondary outcomes of this study will test the additional effects of exercise training on traditional cardiometabolic risk factors [body composition, blood pressure, glycohemoglobin (HbA1c), serum lipids, and insulin resistance] and non-traditional cardiometabolic risk factors (visceral adiposity, endothelial function, cytokines, adipokines, aminotransferases, inflammatory biomarkers, uric acid, ferritin, albumin, and cardiorespiratory and muscular fitness). Also, we include neuronal function biomarkers as measured by neurotrophin-3 (NT-3), neurotrophin-4 (NT-4), and brain-derived neurotrophic factor (BDNF) levels (second secondary objective). Finally, attention capacity and psychological measurements will be assessed to evaluate the additional effect of the exercise intervention on the executive function, quality of life, self-esteem, and well-being of overweight and obese participants. Other covariables of interest include pubertal development, physical activity levels, sedentary behavior, dietary assessment, demographic characteristics, and side effects (Table 2).

**Table 2:** Overview of the measurements and methodology at baseline and post-test in the HEPAFIT study

Measure	Methodology
Primary outcome	
Hepatic fat (%)	Vibration-controlled transient elastograph
Secondary outcomes	
Body mass (kg)	Scale
Height (cm)	Stadiometer
Waist circumference (cm)	Non-elastic tape
Blood pressure	Oscillometric monitor device
Heart rate	Oscillometric monitor device
Lean mass (kg), total body fat (%), bone mineral density (g/cm^2^), abdominal adiposity (g), truncal adiposity (g), limb adiposity (g)	Dual X-ray absorptiometry
Visceral adiposity (cm^2^)	Bioelectrical impedance
High-density lipoprotein cholesterol (mg/dL), low-density lipoprotein cholesterol (mg/dL), total cholesterol (mg/dL), total triglycerides (mg/dL), serum glucose (mg/dL)	Enzymatic spectrophotometry
Serum insulin (mU/L)	Electrochemiluminescence immunoassay
Glycohemoglobin (mg/dL)	Electrochemiluminescence immunoassay
Alanine (U/L), aspartate (U/L), gamma-glutamyl transferase (U/L)	Enzymatic assay
Uric acid, albumin (mg/dL), ferritin (mg/dL)	Enzymatic assay
hs-C reactive protein, ENA-78, GCSF, GM-CSF, GRO, GRO-α, I-309, IL-1α, IL-1β, IL-2, IL-3, IL-4, IL-5, IL-6, IL-7, IL-8, IL-10, IL-12 p40/p70, IL-13, IL-15, IFN-γ, MCP-1, MCP-2, MCP-3, MCSF, MDC, MIG, MIP-1β, MIP-1δ, RANTES, SCF, SDF-1, TARC, TGF-β1, TNF-α, TNF-β, EGF, IGF-I, angiogenin, oncostatin M, thrombopoietin, VEGF-A, PDGF-BB, leptin, BLC, Ckß8–1, eotaxin, eotaxin-2, eotaxin-3, FGF-4, FGF-6, FGF-7, FGF-9, Flt-3 ligand, fractalkine, GCP-2, GDNF, HGF, IGFBP-1, IGFBP-2, IGFBP-3, IGFBP-4, IL-16, IP-10, LIF, LIGHT, MCP-4, MIF, MIP-3α, NAP-2, osteopontin, osteoprotegerin, PARC, PLGF, TGF-β2, TGF-β3, TIMP-1, TIMP-2 (mean spot pixel densities)	Proteome Profiler human cytokine array panel A kit
NT-3, NT-4, BDNF (pg/mL)	Surface plasmon resonance biosensors
Flow-mediated dilation	High-resolution ultrasound device
Carotid intima-media thickness	High-resolution ultrasound device
Cardiorespiratory fitness	20-m shuttle run test Cardiopulmonary exercise test
Muscular strength	Handgrip strength Standing long jump tests
Speed and agility	4 × 10 shuttle run test
Flexibility	Sit-and-reach test
Attention capacity	d2 attention test Stroop task test
Physical activity enjoyment	Physical activity enjoyment scale
Potential confounders	
Physical activity and sedentary behavior	Actigraph GT1M, dual-axis accelerometer ActivPal
Dietary assessment	24-h diet record Food frequency questionnaires
Pubertal development (Tanner stage)	Questionnaire
Family medical history, demographic characteristics, socioeconomic status	Questionnaire

#### Primary outcome

The controlled attenuation parameter is the ultrasonic attenuation coefficient of the ultrasonic signals used during transient elastography examination and is expressed in decibels per meter (dB/m; range from 112 to 242 dB/m), acquired at a 3.5-MHz standard M probe (diameter 7 mm) with a FibroScan® 502 Touch Mode M. Before and during the examination, the adolescent will lie on their back with the right arm in maximum abduction, and abdominal ultrasound will be used to identify a region of the liver free of large vascular structures. The tip of the FibroScan® transducer will then be placed on the skin between the ribs over the right lobe of the liver, and the liver stiffness will be recorded. The technical background and reference values in the pediatric population (1 to 18 years) have been recently described in detail [36]. The algorithm is included in the transient elastography software and data are automatically calculated simultaneously with the liver stiffness measurement.

#### Secondary outcome measures

Blood samples will be obtained from each subject early in the morning by venipuncture from the antecubital vein, following a 10-h overnight fast. Blood samples will be drawn into a tube containing ~1.8 mg EDTA-K3 per mL blood for plasma and a tube with a polymer gel for serum determinations (Vacutainer, Becton Dickinson & Company 2017). Samples will be handled according to Clinical Laboratory Improvement Amendments, which must be followed to achieve valid test results that can be used for diagnoses [37]. Blood samples will be stored at room temperature until centrifugation. Samples should undergo centrifugation immediately. This will be carried out at 1500*g* for 15 min at 4 °C and will give three layers (from top to bottom): plasma, leukocytes (buffy coat), and erythrocytes. The supernatant (plasma) and serum will be carefully aspirated at room temperature and aliquoted into cryovials. Finally, both samples will be stored at −80 °C before the analysis of serum lipids, adipokines, aminotransferases, inflammatory biomarkers, uric acid, ferritin, albumin insulin, and neuronal function biomarkers using surface plasmon resonance biosensors.

Serum lipids, including high-density lipoprotein cholesterol (HDL-C), LDL-C, total cholesterol, and total triglyceride, will be measured with an automatic biochemical analyzer (Deyi Biomedical Technology Co., Ltd., Beijing, China). Concentrations of serum insulin and HbA1c will be analyzed with commercially available electrochemiluminescence immunoassay kits (Roche Diagnostics GmbH, Mannheim, Germany). The homeostatic model assessment of insulin resistance (HOMA-IR) and homeostatic model assessment of pancreatic β-cell function (HOMA-β) indices will be calculated with the following equation:

$$ \mathrm{HOMA}-\mathrm{IR}=\mathrm{fasting}\ \mathrm{insulin}\ \left(\mathrm{FINS};\mathrm{Ins}0\right)\times \mathrm{fasting}\ \mathrm{blood}\ \mathrm{glucose}/22.5 $$

and

$$ \mathrm{HOMA}-\beta =20\times \mathrm{Ins}0/\left(\mathrm{fasting}\ \mathrm{blood}\ \mathrm{glucose}-3.5\right) $$

Alanine aminotransferase, aspartate aminotransferase, and g-glutamyl transferase levels will be determined by enzymatic assays (Beckman Coulter, Brea, CA, United States). Uric acid, albumin, and ferritin will be measured in an enzymatic spectrophotometer (Beckman Coulter, Brea, CA, United States). All assays will be measured in duplicate according to the manufacturers’ standard procedures.

Non-traditional biomarkers will be evaluated for the presence and relative amounts of 80 different inflammatory markers using a Proteome Profiler Human Cytokine Array Panel A kit (R&D Systems, Minneapolis, MN) (Additional file 1: Table S1). These include: hs-C reactive protein, ENA-78, GCSF, GM-CSF, GRO, GRO-α, I-309, IL-1α, IL-1β, IL-2, IL-3, IL-4, IL-5, IL-6, IL-7, IL-8, IL-10, IL-12 p40/p70, IL-13, IL-15, IFN-γ, MCP-1, MCP-2, MCP-3, MCSF, MDC, MIG, MIP-1β, MIP-1δ, RANTES, SCF, SDF-1, TARC, TGF-β1, TNF-α, TNF-β, EGF, IGF-I, angiogenin, oncostatin M, thrombopoietin, VEGF-A, PDGF-BB, leptin, BDNF, BLC, Ckß8–1, eotaxin, eotaxin-2, eotaxin-3, FGF-4, FGF-6, FGF-7, FGF-9, Flt-3 ligand, fractalkine, GCP-2, GDNF, HGF, IGFBP-1, IGFBP-2, IGFBP-3, IGFBP-4, IL-16, IP-10, LIF, LIGHT, MCP-4, MIF, MIP-3α, NAP-2, NT-3, NT-4, osteopontin, osteoprotegerin, PARC, PLGF, TGF-β2, TGF-β3, TIMP-1, and TIMP-2.

This assay will be performed according to the instructions provided by the manufacturer. Briefly, 50–200 μL of serum samples will be thoroughly suspended in 1.5 mL of array buffer 5, incubated at room temperature for 15 min, and centrifuged for 5 min at 5000*g*. The supernatant (1 mL each) will be added to a cocktail of biotinylated antibodies and incubated at room temperature for 1 h. The sample/antibody mixture will be subsequently incubated at 4 °C for 19 h with a membrane embedded with antibodies specific to each of the 80 different inflammatory markers analyzed. The pixel densities of each blot (band), representing the amount of each inflammatory marker present, will be determined using the ImageJ software (National Institutes of Health, Bethesda, MD). These non-traditional biomarkers will be measured in a random sub-sample (*n* = 10, for each group).

Surface plasmon resonance allows real-time monitoring of NT-3, NT-4, and BDNF (R&D Systems, Minnesota, USA). In a typical experiment, the signal reflection, measured at a fixed angular position, is evaluated as a function of time. The real-time quantification will use a specific anti-protein antibody/protein. All experiments will be carried out at 25 °C using a Biacore 2000 instrument with a CM5 chip (Biacore, Uppsala, Sweden). HBS-EB buffer (10 mM 4-(2 hydroxyethyl) piperazine-1-ethanesulfonic acid [HEPES], 150 mM NaCl, 3 mM ethylene diamine tetraacetic acid [EDTA], 0.005% Tween 20, pH 7.4) will be used as a continuous running buffer at a 5–60 μg/mL flow rate. The antibodies (ligand) anti-NT-3, anti-NT-4, and anti-BDNF will be immobilized onto the CM5 sensor surface at a concentration range of 10–50 μg/mL. Before immobilization of the antibodies, the sensor surface will be activated via an amino-coupling chemistry kit [1-ethyl-3-(3′-dimethylaminopropyl) carbodiimide, N-hydroxysuccinimide (GE Healthcare, Uppsala, Sweden)]. For the immobilization steps, the basic principles of assays using surface plasmon resonance will be applied, such as preconcentration and a binding assay with the analyte. Each antibody solution will be injected individually to couple with the activated surface until the appropriate immobilization level is achieved. Activated carboxyl group excess will be blocked by 1 M ethanolamine hydrochloride (pH 8.5). A reference or control flow cell with 50 μg/mL bovine serum albumin (Sigma-Aldrich, Saint Louis, MO) at pH 5.0, but no immobilizing solution, will be used to subtract the instrument systematic noise and drift (background response). After each injection of a binding assay with the analyte and standard samples, an optimal regeneration solution will be injected in agreement with previous studies [38, 39]. The percentage of surface regeneration will be estimated using [1 − (Rreg/Ro) × 100]. If the percentage regeneration achieved is below 10%, other regeneration solutions should be injected until the percentage regeneration is higher than 50%. Standard curves will be constructed by dilution of each standard recombinant protein NT-3, NT-4, and BDNF. A range of 20–5000 ng/mL will be used to obtain the curve. Each sample of protein will be injected in triplicate.

Diluted plasma samples will be quantified and the binding response will be calculated by subtracting the response measured in the control flow cell from the response in the sample flow cell. This value is applied to the simple 1 : 1 L binding model (A + B or AB). The Langmuir model is the most commonly used model for calculating binding affinity. The report points will be taken 10 s before and 100 s after taking a measurement and the difference between these two report points will be taken as the end-point measurement.

##### Endothelial function

Endothelial function may potentially represent a quantitative method to assess the presence and extent of childhood cardiometabolic risk before the development of overt disease [40]. Flow-mediated dilation will be measured with the technique introduced by Ramírez-Vélez [41] in the Colombian population, using the guidelines reported by Corretti et al. [42]. The diameter of the brachial artery will be assessed using a high-resolution ultrasound device (Mindray M-9®, Shenzhen, China), equipped with a 7.5–10 MHz linear array transducer and an integrated electrocardiography package. B-mode images were obtained at a reproducible point in the distal third of the upper arm. Endothelium-dependent vasodilation was assessed by measuring flow-mediated dilation after 5 min of forearm ischemia [43]. All measurements will be taken at the end of diastole as observed by electrocardiogram (ECG). Briefly, a 1-min baseline measurement will be taken and then a pneumatic rapid cuff inflator (Hokanson, Bellevue, WA), placed around the forearm distal to the humeral epicondyle, will be inflated to 220 mmHg for 5 min. Recording of the image will be ceased on inflation of the cuff and recommenced 30 s before deflation. Peak dilation during each study will be defined as the greatest percentage change from the resting baseline brachial artery diameter. The intra-session coefficients of variations will be ≤1% for the baseline diameter. Reliability, estimated by intra-class correlation coefficients based on four baseline measurements (*n* = 8 subjects), showed an intra-class correlation coefficient of 0.91 for the baseline diameter and 0.83 for flow-mediated dilation (unpublished observations). Images will be recorded on a DVD player for subsequent measurements by one observer blinded to the study.

##### Carotid intima-media thickness

The carotid intima-media thickness (cIMT) will be assessed using semi-automated B- and M-mode ultrasound, (Mindray M-9®, Shenzhen, China) with a high-frequency linear-array probe (5–13 MHz) [44]. In brief, the left and right common carotid arteries will be imaged at a standardized magnification (2 × 2 cm). A participant’s head will be turned 45° away from the site being scanned. Optimal images will be captured at the end of diastole, incident with the R-wave of the ECG, for later analysis. Our inter-observer intra-class coefficient for cIMT measurements is 0.99 (coefficient of variation 0.99%) and the intra-observer intra-class coefficient for cIMT measurements is 0.98 (coefficient of variation 1.9%) (unpublished observations).

##### Anthropometric measures

Variables will be collected at the same time in the morning, between 7:00 and 10:00 a.m., following an overnight fast. The body weight of the subjects will be measured when the subjects are in their underwear and not wearing shoes, using electronic scales (Tanita® BC544, Tokyo, Japan). Height (using a stadiometer, Seca® 213, Hamburg, Germany), waist circumference, and hip circumference will be measured in centimeters using a tape measure (Ohaus® 8004-MA, Parsippany, NJ, USA). The waist circumference is measured as the smallest circumference around the torso between the end of the xiphisternum and the top of the iliac crests. The hips are measured at the greatest circumference between the iliac crests and the upper femur. Body mass index (BMI) will be calculated as the body weight in kilograms divided by the square of the height in meters. BMI will be classified as underweight, normal weight, overweight, or obese using the World Health Organization criteria [45]. The anthropometric measurements will be conducted by a nutritionist in accordance with International Society for the Advancement of Kinanthropometry guidelines [46]. The mean of three consecutive measurements will be recorded.

##### Body composition

We will also measure fat mass, lean body mass, and subcutaneous abdominal fat by conducting a dual-energy X-ray absorptiometry (DEXA) scan (HOLOGIC, QDR 4500 W). Visceral fat volume will be measured by bioelectrical impedance. An experienced DEXA technologist blinded to the study randomization will perform the DEXA imaging studies. The mean precision of our machine is 1.1% for soft tissue (unpublished observations).

##### Physical fitness

Physical fitness includes cardiorespiratory fitness, muscular strength, speed and agility, and flexibility. Moreover, physical fitness has been reported as a powerful marker of health in childhood, and its inclusion in the definition of metabolic syndrome has been proposed [47]. Cardiorespiratory fitness will be measured using the 20-m shuttle run test as previously described by Léger et al. [48]. The cardiorespiratory profile will be derived by computing standardized values of the number of completed laps performed by each participant by age and sex. Upper-body isometric strength (handgrip strength test) will be assessed using a handgrip dynamometer, (T.K.K. 5001, Grip-A, Takei, Japan), adjusted by sex and hand size for each adolescent [49]. The participants will be instructed to stand with their arms completely extended, squeezing the handgrip gradually and continuously up to the maximum of their strength, for at least 2 s, performing the test twice, alternating hands. The best score for each hand will be recorded in kilograms. The handgrip score (kg) will be calculated as the average of the left and right grip strength and then expressed per kilogram of body weight [50]. Lower-body explosive strength (standing long jump test) will be tested in an indoor gymnasium with a wooden floor. The adolescents will be instructed to push off vigorously from the starting line to jump as far forward as possible while landing on both feet and staying upright [51]. The test will be done twice, and the best attempt will be recorded. The standing-jump score will be determined by the distance between the last heel mark and the take-off line. The results of the handgrip strength and standing long jump tests will be transformed into standardized values (*z* scores) by age and sex. The *z* scores of the two tests will be added together to yield the muscular fitness score. Speed and agility (movement speed, coordination, and agility assessment) will be measured using the 4 × 10 shuttle-run test. The time that it takes to complete the test will be recorded to the nearest tenth of a second [52]. Flexibility will be measured according to the standard sit-and-reach test for range of movement (in cm).

##### Resting blood pressure and heart rate

An automated device will measure resting HR and blood pressure. Participants will be instructed to rest for 10 min prior to the measurement. HR and blood pressure will be measured twice from the dominant arm while the subject is in a supine position. This will occur at the initial screening visit and 30 min after training.

##### Attention capacity

The d2 test of attention is a paper and pencil test that consists of 14 different lines of 47 randomly mixed letters (“p” or “d”). Briefly, for each line, the adolescents will be given 20 s to identify and mark the letter “d” when it is presented with two dashes placed above and/or below it (i.e., relevant elements). The remaining combinations are considered irrelevant elements. After 20 s, there is an acoustic signal, which prompts the participants to progress to the next line. Their attention capacity will be calculated as the number of correct relevant elements minus the number of irrelevant elements marked. The score will be transformed into age-specific percentiles. Thus, a higher attentional ability is indicated by a higher score [53].

##### Stroop task test

We will use the New Stroop Test II (Spanish version). This test measures selective attention, cognitive flexibility, and processing speed. It is used to evaluate executive function [54]. The test consists of two control tasks: a Stroop task and a reverse Stroop task. The number of correct answers, reverse-Stroop interference rate, and Stroop interference rate will be calculated.

##### Physical activity enjoyment scale

Before, during, and immediately upon completion of each protocol, participants will also rate their perceived enjoyment according to the physical activity enjoyment scale, which has been validated by Kendzierski and DeCarlo [55]. Each item is rated on a seven-point bipolar scale with four representing a neutral point in terms of how much the respondent enjoyed the exercise.

##### Physical activity and sedentary behavior

The Actigraph GT3x dual-axis accelerometer (Actigraph, Pensacola, FL, USA) and ActivPAL monitor (PAL Technologies Ltd., Glasgow, UK) will be used. The Actigraph GT3x measures vertical accelerations from 0.05*g* to 2*g*, sampled at 30 Hz, at 5-s epoch intervals. The ActivPAL summarizes data in 15-s epoch intervals over 24 h at a sampling frequency of 10 Hz. Before the beginning of each MOVI-2 program session, the accelerometer will be positioned at the waist in line with the right axilla, at the level of the iliac crest of the right hip and secured by an elastic belt round the hip. The Actigraph GT3x has been validated for estimating energy expenditure during physical activity in a youth population [56]. Data from the accelerometer will be used to calculate the average counts/min for each game. Accelerometers will also be worn continuously for seven consecutive days to record physical activity intensity levels and patterns, as well as sleeping habits. Participants will also complete a diary log. Data stored in the device will be analyzed using the ACTi-life 6.01 software.

##### Dietary assessment

To determine the average habitual energy and macronutrient intake, a detailed 24-h diet record will be obtained from each adolescent for one weekday and one weekend day during the 1-week baseline period. Food intake analysis software (FAO/INFOODS, Report of the Technical Workshop on Standards for Food Composition Data Interchange, Rome 2004) and national food composition tables (for specific foods) will be used to analyze the total energy and macronutrient intake of each subject’s 24-h diet. The dietary assessment will be conducted by a registered nutritionist.

##### Sociodemographic information

Baseline sociodemographic values (sex, highest educational level attained by mother/father, composition of household, others), which could act as covariates or confounders for the tested treatment modality, will also be collected. The surveys will include questions on age, education, occupation, income, health history, and alcohol consumption, among others.

##### Side effects and monitoring

The study will be conducted according to good clinical practice and standard operating procedures. It will be monitored by the Human Rights Committee at the Universidad del Rosario Coordinating Centre, a committee composed of experts in physical exercise and sports medicine, plus physical therapists, physical educators, and clinical epidemiologists. Interim monitoring reports will be submitted to the experts, focusing on patient intake, adherence to the protocol, baseline comparability of treatment groups, completeness of data retrieval, and adverse events. All adverse events will also be reported to the Universidad del Rosario Ethics Committee. To standardize the study procedures, an operations manual has been written, and comprehensive training sessions will be held prior to initiation of the trial. An independent researcher will be in charge of auditing all assessment staff to record all of these events for the participants over the study period.

### Power calculation and sample size

Change in liver fat is the primary outcome of this study. For liver fat measured by a FibroScan 502®, a sample size of 100 will have 80% power to detect a difference in hepatic fat of 5.0% assuming a standard deviation of 4.1% and an α of 0.025 for a two-sided significance level. Assuming a maximum a loss of follow-up of 10%, we decided to recruit a total of 100 subjects, 25 participants for each group. We assume that the dropout rate will be similar to that in our previous studies performed in a young overweight population with a similar age range and a similar intervention program [18, 23–25, 57–59]. The sample size will allow for multivariate analyses with up to three predictors, assuming moderate size effects.

### Blinding and randomization

Each participant will have a 0.25 probability of being assigned to any study group. This assignment will be made through previously constructed balanced blocks. These blocks permit an equi-probabilistic assignment and ensure that each group has the same number of participants. This assignment mechanism will continue until all 100 participants have been assigned to their interventions. The study’s health personnel will act independently of the personnel in the school from where the participants are selected. Therefore, they do not carry out any school activities and their tasks are limited to collecting data for this study. The assignment codes will be maintained in a confidential file in the research center of the coordinating group and will be opened when the study analysis is finished. At the beginning and end of their participation in the study, the primary and secondary outcomes will be determined for each of the patients.

Given that physical exercise is an intervention that cannot be masked from the participants or researchers, professionals who are unaware of each subject’s group assignment will perform the measurement and analysis of the resulting variables. To ensure masking, randomization will be applied through balanced blocks and an alphabetical code will be assigned to each study group. This code will be in the custody of the administrative coordinator of the research group and will not be revealed to the professionals in charge of processing the data until the analysis with coded interventions has terminated.

### Adherence and compliance

The CEMA research center is responsible for preparing the reports for the safety committee to audit the study’s progress. These reports will include the following information: (a) recruitment and follow-up per person per month and comparison with the pre-established goals, (b) adherence of participants (biochemical markers and data on the degree of compliance and a comparison with the objectives foreseen for the exercise groups, (c) quality control (preferences for certain digits, variability, and values out of range), and (d) intermediate and final, primary, and secondary variables for each group and comparisons among groups with respect to the primary variables and other secondary variables. For the interventions with additional exercise, 75% adherence is expected of the 72 planned sessions, along with at least 70% training compliance.

### Data quality

To evaluate the veracity of the data from the questionnaires and from the database, an external personnel team assigned to each group will revise the instruments and questionnaires for a random number of participants. The proposal is to review the data from approximately 25% of the participants in each group, and CEMA members will confirm and certify the validity of the data. The CEMA researchers will create a system to access the study data through a private website (http://www.urosario.edu.co/cema/inicio/), from where authorized researchers can download all the forms, databases, and articles published. For security and privacy, a valid ID and password are necessary to access the data and the forms. This web-based system is used to send data to the data manager. As a quality control measure, the principal study data will be reviewed for features such as a preference for certain figures and data variability. All the forms will be entered in duplicate and they will be checked for inconsistencies and missing data. To minimize potential error, a *Manual of Operations* has been drafted. Until the end of the trial, personnel from the groups will have access to all the information from the trial, except for the statisticians and the safety committee.

### Statistical analysis

The final data will be analyzed using IBM SPSS 22.0 (SPSS, Inc., Chicago, IL, USA) and SAS software (SAS Institute Inc., Cary, NC, USA). An exploratory analysis will be performed to determine the frequency, range, variability, and distribution type for each variable to establish the most appropriate statistical test when comparisons are necessary. These analyses will assess the primary outcome (hepatic fat) and will be undertaken using the intention-to-treat principle. The intention-to-treat analysis for this study will include all participants, including those who are not fully compliant and those with missing outcome data.

Because this is an experimental design with the primary and secondary outcomes measured twice, the first at baseline (*t*_0_ = 0 months) and the second after the interventions (*t*_1_ = 6 months), for four study groups, a comparative analysis between the measurements will determine the differences. A linear mixed model with repeated observations will be used for these comparisons, when appropriate, for each dependent variable. Subsequently, a multivariate analysis will be carried out, and the autocorrelation between repeated measures will be taken into account. We will use longitudinal analysis methods, such as a generalized estimating equation approach, to control the differences among measurements at baseline and to incorporate incomplete observations into this analysis. Pearson correlations will be calculated between the maximal values of the primary and secondary outcomes. Finally, we will investigate whether there is an interaction between the two interventions for the primary outcome after 30–40 min of supervised HIPE and LIPE or combined training (HIPE + LIPE). For these analyses, we will include appropriate interaction terms in the models [60]. We plan to report regression coefficients for the interaction terms and their 95% confidence intervals.

## Discussion

The relevance of this project is that the results obtained from the objectives proposed will contribute to our understanding of the links between excess weight and alterations in hepatic and adipose metabolism. Moreover, we may be able to identify biomarkers for metabolic abnormalities associated with central obesity that are involved in the induction of oxidative dysfunction processes via lipotoxicity, which is common in physical inactivity and obesity, such as pathologies related to the cardiometabolic system. The study of biomarker profiles in adolescents with metabolic syndrome and obesity compared to a normal weight population could assist in the early diagnosis of cardiovascular diseases. Future studies are warranted to verify the relevance of the biomarkers associated with obesity and elucidate the underlying biochemical processes.

Currently, the management of overweight conditions and public information on the topic have focused on treatments such as caloric restriction, physical exercise, and different drugs. There have been promising results, due to the distinct metabolic adaptations that can prove beneficial for individuals with excess weight. Thus, our research hypothesis is that some physical exercise programs will generate a higher reduction of fat in liver than other interventions. In addition, there may be improvements in the hepatic metabolism and cardiovascular health of overweight/obese adolescents from the district of Bogotá.

At a clinical level, interventions to prevent excess weight and sedentary habits are very costly and the results in the pediatric setting are discouraging and not very cost-effective [60–62]. Hence, our research seeks to evaluate the impact of the interventions, and whether they are easy to implement, safe, economic, and free of secondary effects. The research focuses on assessing hepatic metabolic activity (fat content, as a primary outcome) and bodily composition, blood pressure, insulin sensitivity, lipid and carbohydrate metabolism, and physical condition (secondary outcomes), which are highly relevant.

Because adolescence is a critical period in the selection of and preferences for particular forms of conduct and behavior, it is necessary to promote strategies that help in strengthening healthy habits during this time of life. We postulate that interventions such as supervised physical exercise aimed at preventing metabolic dysfunction, which is characterized by excess weight and sedentary habits, could diminish the incidence or progression of non-chronic disease. The aforementioned hypothesis is based on solid evidence, which shows that engaging regularly in vigorous physical exercise for at least 12 weeks improves physical condition [63, 64], body composition [65], and most cardiovascular risk factors [19]. However, even though they are safe interventions [18, 19], the effect of different exercise intensities, and in particular, high-intensity work, on liver metabolism is unknown [66].

### Dissemination

We will disseminate the results of our study via presentations at international conferences and articles in peer-reviewed journals. The study will be implemented and reported in accordance with the SPIRIT guidelines (Standard Protocol Items: Recommendations for Interventional Trials) [67] (Additional file 2).

### Future directions

In the short term, this project offers the opportunity to obtain detailed measurements of body composition, cardiometabolic health, and hepatic function in overweight children and adolescents. It will yield information on the effects of physical exercise on them. In the long term, we expect that the results obtained will deepen our knowledge of the effects of supervised physical exercise in the school environment on some of the most frequent metabolic disorders, as well as validate strategies that promote actions aimed at mitigating the risk of the occurrence of these disorders and reducing morbidity. Both of the latter are seen as priority problems by the Ministry of Health and Social Protection. Our work lies within the normative framework for generating preventive alternatives and effective therapeutics described in Colombia’s Integral Health Information System and the Public Health Surveillance System.

### Trial status

The recruitment of adolescents started in January 2018 and will continue through the end of December 2018.

## Additional files

Additional file 1: Non-traditional biomarkers. (DOCX 19 kb)

Additional file 2: SPIRIT 2013 Checklist: Recommended items to address in a clinical trial protocol and related documents. (DOC 125 kb)

## Abbreviations

A1AT: Alpha-1 antitrypsin
BDNF: Brain-derived neurotrophic factor
BMI: Body mass index
CEMA: Centre for Studies of Physical Activity Measurements (in Spanish)
cIMT: Carotid intima-media thickness
DEXA: Dual-energy X-ray absorptiometry
ECG: Electrocardiogram
EDTA: Ethylene diamine tetraacetic acid
HbA1c: Glycohemoglobin
HDL-C: High-density lipoprotein cholesterol
HIIT: High-intensity interval training
HIPE: High-intensity physical education
HOMA-IR: Homeostatic model assessment of insulin resistance
HOMA-β: Homeostatic model assessment of pancreatic β-cell function
HR: Heart rate
HRmax: Maximum heart rate
LDL-C: Low-density lipoprotein cholesterol
LIPE: Low-to-moderate intensity physical education
MET: Metabolic equivalent
MICT: Moderate-intensity continuous training
MOVI-2: Physical Activity Intervention on the Obesity of Schoolchildren (in Spanish)
NAFLD: Non-alcoholic fatty liver disease
NT: Neurotrophin
