# Supplementary material for: Effects of an exercise program on hepatic metabolism, hepatic fat, and cardiovascular health in overweight/obese adolescents from Bogotá, Colombia (the HEPAFIT study): study protocol for a randomized controlled trial
Source: Trials. 2018 Jun 25;19:330. doi: 10.1186/s13063-018-2721-5 (PMC6019229; doi:10.1186/s13063-018-2721-5)
Supplement: Supplementary file 1 — Non-traditional biomarkers. (DOCX 19 kb) [file 13063_2018_2721_MOESM1_ESM.docx]

**Additional file 1**

| **GCSF:** Granulocyte-colony stimulating factor |
| --- |
| **ENA-78:** Epithelial-derived neutrophil-activating peptide 78 |
| **GM-CSF:** Granulocyte-macrophage colony-stimulating factor |
| **GRO:** Protein groucho |
| **GRO-alpha:** Growth-regulated alpha protein |
| **I-309:** I - 309 recombinant protein |
| **IL-1alpha:** Interleukin 1 alpha |
| **IL-1beta:** Interleukin 1 beta |
| **IL-2:** Interleukin 2 |
| **IL-3:** Interleukin 3 |
| **IL-4:** Interleukin 4 |
| **IL-5:** Interleukin 5 |
| **IL-6:** Interleukin 6 |
| **IL-7:** Interleukin 7 |
| **IL-8:** Interleukin 8 |
| **IL-10:** Interleukin 10 |
| **IL-12 p40/p70:** Interleukin 12 p40/p 70 |
| **IL-13:** Interleukin 13 |
| **IL-15:** Interleukin 15 |
| **IFN-gamma:** Interferon gamma protein |
| **MCP-1:** Monocyte Chemoattractant Protein-1 |
| **MCP-2:** Monocyte Chemoattractant Protein-2 |
| **MCP-3:** Monocyte Chemoattractant Protein-3 |
| **MCSF:** Macrophage colony-stimulating factor |
| **MDC:** Macrophage–derived Chemokine |
| **MIG:** Monokine induced by interferon-gamma |
| **MIP-1beta:** Macrophage Inflammatory Proteins-1beta |
| **MIP-1delta:** Macrophage Inflammatory Proteins-1delta |
| **RANTES:** regulated on activation, normal T cell expressed and secreted |
| **SCF:** Skp, Cullin, F-box containing complex |
| **SDF-1:** Stromal cell-derived factor 1 |
| **TARC:** Thymus and activation regulated chemokine |
| **TGF-beta1**: Transforming Growth Factor-β1 |
| **TNF-alpha:** Tumor necrosis factor- alpha |
| **EGF:** Epidermal growth factor |
| **IGF-I:** Insulin-like growth factor I |
| **Ang:** Angiogenin |
| **OSM:** Oncostatin M |
| **THPO:** Thrombopoietin |
| **VEGF-A:** Vascular endothelial growth factor A |
| **PDGF-BB:** Platelet-derived growth factor-BB |
| **Leptin** |
| **BDNF:** Brain-derived neurotrophic factor |
| **BLC:** B lymphocyte chemoattractant |
| **Ckß8-1:** CC chemokine (CCL23) |
| **Eotaxin-1:** CC chemokine (CCL11) |
| **Eotaxin-2:** CC chemokine (CCL24) |
| **Eotaxin-3:** CC chemokine (CCL26) |
| **FGF-4:** Fibroblast growth factor 4 |
| **FGF-6:** Fibroblast growth factor 4 |
| **FGF-7:** Fibroblast growth factor 4 |
| **FGF-9:** Fibroblast growth factor 4 |
| **Flt-3 Ligand:** Flt3 ligand human recombinant |
| **Fractalkine:** Chemokine (C-X3-C motif) ligand 1 |
| **GCP-2:** Granulocyte chemotactic protein-2 |
| **GDNF:** Glial cell line derived neurotrophic factor |
| **HGF:** Hepatocyte growth factor protein |
| **IGFBP-1:** Insulin like growth factor binding protein 1 |
| **IGFBP-2:** Insulin like growth factor binding protein 2 |
| **IGFBP-3:** Insulin like growth factor binding protein 3 |
| **IGFBP-4:** Insulin like growth factor binding protein 4 |
| **IL-16:** Interleukin-16 |
| **IP-10:** Interferon gamma-induced protein 10 |
| **LIF:** Leukemia inhibitory factor |
| **LIGHT:** Lymphotoxin, exhibits Inducible expression and competes with HSV Glycoprotein D for binding to Herpesvirus entry mediator, a receptor expressed on T lymphocytes |
| **MCP-4:** Potential role for monocyte chemotactic protein-4 |
| **MIF:** Macrophage migration inhibitory factor |
| **MIP-3:** Macrophage inflammatory protein-3 |
| **NAP-2:** Neutrophil-activating peptide |
| **NT-3:** Neurotrophin-3 |
| **NT-4:** Neurotrophin-4 |
| **OPG:** Osteoprotegerin |
| **OPN:** Osteopontin |
| **PARC:** Cytoplasmic anchor protein in p53 |
| **PLGF:** Placental growth factor |
| **TGF-beta2:** transforming growth factor beta 2 |
| **TGF-beta3:** transforming growth factor beta 3 |
| **TIMP-1:** Tissue inhibitor of metalloproteinases-1 |
| **TIMP-2:** Tissue inhibitor of metalloproteinases-2 |
